# A Novel In Vitro Model for Studying Quiescence and Activation of Primary Isolated Human Myoblasts

**DOI:** 10.1371/journal.pone.0064067

**Published:** 2013-05-23

**Authors:** Jeeva Sellathurai, Sirisha Cheedipudi, Jyotsna Dhawan, Henrik Daa Schrøder

**Affiliations:** 1 Institute of Clinical Research, SDU Muscle Research Cluster (SMRC), University of Southern Denmark, Odense, Denmark; 2 Max Planck Institute for Heart and Lung Research, Bad Nauheim, Germany; 3 Institute for Stem Cell Biology and Regenerative Medicine (InStem), National Centre for Biological Sciences, Tata Institute of Fundamental Research, Bangalore, India; 4 Department of Clinical Pathology, Odense University Hospital, Odense, Denmark; Stem Cell Research Institute, Belgium

## Abstract

Skeletal muscle stem cells, satellite cells, are normally quiescent but become activated upon muscle injury. Recruitment of resident satellite cells may be a useful strategy for treatment of muscle disorders, but little is known about gene expression in quiescent human satellite cells or the mechanisms involved in their early activation. We have developed a method to induce quiescence in purified primary human myoblasts isolated from healthy individuals. Analysis of the resting state showed absence of BrdU incorporation and lack of KI67 expression, as well as the extended kinetics during synchronous reactivation into the cell cycle, confirming arrest in the G_0_ phase. Reactivation studies showed that the majority (>95%) of the G_0_ arrested cells were able to re-enter the cell cycle, confirming reversibility of arrest. Furthermore, a panel of important myogenic factors showed expression patterns similar to those reported for mouse satellite cells in G_0_, reactivated and differentiated cultures, supporting the applicability of the human model. In addition, gene expression profiling showed that a large number of genes (4598) were differentially expressed in cells activated from G0 compared to long term exponentially proliferating cultures normally used for in vitro studies. Human myoblasts cultured through many passages inevitably consist of a mixture of proliferating and non-proliferating cells, while cells activated from G_0_ are in a synchronously proliferating phase, and therefore may be a better model for in vivo proliferating satellite cells. Furthermore, the temporal propagation of proliferation in these synchronized cultures resembles the pattern seen in vivo during regeneration. We therefore present this culture model as a useful and novel condition for molecular analysis of quiescence and reactivation of human myoblasts.

## Introduction

Tissue specific stem cells are present in many adult tissues. In bone marrow and epithelia, the stem cell population is continuously active and maintains the homeostasis of the tissues [1]–[4], while in skeletal muscle, the tissue specific stem cells (satellite cells) are normally quiescent but can be recruited after an injury. Due to the presence of satellite cells (SC), muscle has a considerable capacity for regeneration. In intact muscle, the quiescent SC is situated between the basement membrane and the muscle fiber. In response to damage, the differentiated myofibers experience injury and degenerate, but the SCs are activated from G_0_ and enter the cell cycle. Most of the resulting myoblasts continue into differentiation, fuse and form new muscle fibers, but a small minority returns to G_0_ and restore the resting SC compartment [5]–[8]. The whole regeneration process is completed in less than three weeks [9]. While the mechanisms regulating proliferation and differentiation have been widely studied, the mechanisms involved in exit from and entrance into, and maintenance of the quiescent state, G_0_, are less well understood, particularly in the context of human muscle. However, from a biological perspective the G_0_ transition, activation and preservation of the stem cell niche depend on a balance between inducing and inhibiting factors [10]. From a therapeutic perspective, the activation from G_0_ and recruitment of resident SC might provide better treatment strategies in various forms of primary myopathies. Even the more common form of muscle weakness seen in sarcopenia, inactivity and prolonged bed rest due to surgery or illness, especially in elderly, might be treatment targets as these conditions involves muscular atrophy resulting in loss of muscle mass and strength [11]–[14].

Considering the large volume of human muscle tissue, stem cell transplantation is unlikely to provide effective treatment of generalized myopathic disorders or sarcopenia. Focus in regenerative medicine therefore has been on intervention aiming at boosting the activated myogenic stem cells and enhance muscle growth [15]–[21]. An alternative target might be activation or recruitment of the SC population; there have been reported benefits concerning muscle strength and endurance due to physical training for immobilized patients [11]–[13], [22], [23] and patients with myopathies [24–26]. Indeed, satellite cell activation is part of this training response. Since SC activation is emerging as a serious alternate target for therapeutic intervention, it is crucial to unravel the molecular mechanisms governing their quiescence and activation.

Analyses of SC activation studies are difficult to conduct in vivo, since SCs only constitute ∼2% of the cells in adult muscle. Various in vitro models have therefore been employed to reduce the complexity of the milieu and increase the SC fraction. Freshly isolated primary SCs are a possible source for such studies, but the number of cells obtained is relatively low and the isolation process itself triggers activation. Low expression of MyoD in freshly isolated cells has been taken to indicate quiescence in some studies [27,28], [27,28]. Single muscle fiber isolation provides another possibility to study the activation of SC in mouse and though the method has been applied to human muscle, it is difficult to obtain intact myofibers [29–31]. Single muscle fibers are excellent for immunocytochemical studies of SC activated while still in association with the fiber, but do not allow study of entry into quiescence.

Thus, experimental studies on quiescent human myoblasts require a model where a large number of cells can be arrested in G_0_ and subsequently reactivated as homogeneous synchronized populations. Such a model has previously been described and employed to study mechanisms in quiescence and activation in the mouse myoblast cell line C2C12 [10,32–36]. Here we have adapted the protocol to human myoblasts and present a model for G_0_ arrest of cultured, proliferating primary isolated human myoblasts. As a major advantage this model enables the study of gene expression in G_0_ arrested and synchronously reactivated human myoblasts as well as during myoblast entry into G_0_, studies that previously were not possible. Our observations form the basis for a new understanding of the resting state in human muscle stem cells.

## Materials and Methods

### Ethics Statement

The participants included in this study gave written informed consent and the local ethics committee of Region of Southern Denmark (S-20070079) approved the study.

### Establishment of Primary Cell Cultures

The three human primary myoblast cultures used in this study was established as previously described [37] with some modifications. Primary cell culture A and B was established from muscle biopsies taken from m. vastus lateralis of two males (18 and 20 years old) and cell culture C was established from muscle tissue from gluteus maximus of a female (18 years old).

### Isolation and Propagation of Human Myoblasts

Biopsies free of connective tissue were minced, washed and dissociated with 0.05% trypsin-EDTA (Invitrogen) for 3×30 min. Harvested mononuclear cells were pooled and Fetal Bovine Serum (FBS, Invitrogen) added as protease inhibitor. The isolated cells were seeded (max 7 passages) on flasks (NUNC) coated with extracellular matrix (ECM, Sigma-Aldrich) and during every passage the cells were preplated for 15 min. in non-coated dishes before transfer to growth medium, GM (DMEM w. 10% FBS and 1% penicillin and streptomycin (PS), Invitrogen).

### G_0_ arrest, Reactivation and Differentiation of Human Myoblast Cultures

The procedure for G_0_ arrest of human myoblasts was modified from previous studies using mouse myoblasts [33]. Proliferating human myoblasts were detached with 0,05% trypsin-EDTA, pre-plated and transferred to semi-solid suspension medium, SM, (DMEM with 2% methyl cellulose (Sigma-Aldrich), 10% FBS and 1% PS) and cultured in dishes with ultra low attachment surface (Corning) with a density of 1.5×10^5^ cells/ml. The loss of substrate attachment in suspension culture triggers the cells to enter G_0_ arrest.

Cells were harvested from suspension medium by 2 rounds of centrifugation after dilution with 4 volumes of PBS. The recovered cells were resuspended in lysis buffer (for RNA isolation), cytospun (for immunocytochemistry) or plated on ECM-coated dishes or coverslips in growth medium for 1–4 days for analysis of reactivation. For analysis of differentiation, G_0_ cells were first cultured in growth medium followed by a shift to differentiation medium, DM (DMEM with 2% FBS, 1% PS and 25 pmol Insulin (Actrapid from Novo Nordisk) for 5–7 days.

### BrdU Incorporation

DNA synthesis was determined by incorporation of 5-bromo-2-deoxyuridine, BrdU (Sigma- Aldrich). For 1-hour pulse BrdU incorporation, cells were incubated in medium with 100 µM BrdU. For cumulative BrdU incorporation, cells were incubated in medium with 10 µM BrdU and harvested after various times. Incorporated BrdU was detected using anti-BrdU antibody.

### Immunocytochemistry

Cells cultured on coverslips (Thermanox from NUNC) in GM and DM were rinsed twice in PBS. G_0_ arrested cells were washed as previously described, re-suspended in PBS, loaded in Shandon cytofunnel (Thermo Scientific) and spun onto SuperFrost® Microscope Slides with Shandon Cytospin 4 Cytocentrifuge.

For detection of Desmin, samples were fixated in acetone, 10 min., followed by addition of mouse-anti-desmin, D-ER-11 (Dako, Denmark) 1∶25. For detection of KI67, samples were incubated in 4% formaldehyde for 15 min. followed by incubation in 96% ethanol for 10 min. Samples were then rinsed in water before heat-induced epitope retrieval for 15 min in Tris-EGTA buffer, pH 9,0 at 95°C, followed by addition of mouse-anti-KI67, MIB1 (Dako) 1∶200. EnVision (Dako) and DAB^+^ was used to detect Desmin and KI67.

For detection of MYH8 (Myosin Heavy Chain 8, neonatal), samples were incubated in mouse-anti-MYH8, WB-MHCN (Novocastra) 1∶10. For detection of Fast Myosin, samples were incubated in mouse-anti-MHC, MY32 (Abcam) 1∶8000. For detection of P53 samples were incubated 15 min. in 4% formaldehyde, then in Triton-X100 for 5 min followed by incubation in mouse-anti-P53 (Novocastra) 1∶200. SGCA was detected by incubation in mouse-anti-SGCA (Novocastra) 1∶20. For detection of FGFR1, samples were incubated in 4% formaldehyde for 10 min. followed by anti-mouse-FGFR1 (Dako) 1∶100 over night. For detection of Myogenin, samples were incubated in 4% formaldehyde for 15 min. followed by incubation in 96% ethanol for 10 min. Samples were then rinsed in water before heat-induced epitope retrieval for 15 min in Tris-EGTA buffer, PH 9,0 at 95°C, followed by addition of mouse-anti-Myogenin, F5D, (Dako), 1∶800. For detection of BrdU the samples were incubated in 2 N HCL with 0.5% Triton-X100 and 0.5% Tween20 for 30 min in order to denature the DNA. Samples were then rinsed 3×5 min in NaBH_4_ solution (1 mg NaBH_4_/ml water) followed by addition of mouse-anti-BrdU, Bu20a (Dako) 1∶20. PowerVision (Dako) and DAB^+^ was used to detect MYH8, Fast Myosin, Myogenin, P53, FGFR1, SGCA and BrdU. Nuclei were counterstained with Mayers Hemalum.

### Morphometric Analyses

CAST version 2.1.6.0 (Visiopharm, Denmark) was used for the morphometric analyses. Random counts were performed including the entire cell containing area. KI67 positive cells were counted in 10% of the area, total cells in 5%. BrdU positive cells and total number of cells were counted in 10% of the sample area. Only cells with nuclear staining were included. The identity of all the samples was blinded.

### RNA Isolation

Cultured cells were rinsed twice in PBS and lysed in 1× Nucleic Acid Purification Lysis Solution (Applied biosystems, Foster City, CA, USA). Samples from suspension cultures were collected at various time points, washed twice in PBS and lysed with Lysis Solution. RNA was isolated using ABI PRISM™ 6100 Nucleic Acid PrepStation with Total RNA Chemistry kit (Applied Biosystems, Foster City, CA, USA) according to the manufacturer’s protocol.

### Real-time Reverse Transcription PCR

RNA was isolated from human myoblast cultures during proliferation, G_0_ arrest, reactivation and differentiation. cDNA was generated from 500 ng of isolated total RNA using High Capacity cDNA Reverse Transcription Kit (Applied Biosystems). qPCR was performed on ABI Prism 7900HT Sequence Detection System using TaqMan Array platform (Applied Biosystems). TaqMan Arrays were custom-designed 384-well micro fluidic cards containing 32 genes including 6 reference genes. All assays were run in triplicates and the experiment series was made with cells from three individuals (3 biological replicates). Raw data was retrieved using the SDS 2.1 software, analyzed with automatic threshold settings and the Cq values were exported to qbase^PLUS^ software (Biogazelle). The most stable reference genes were selected by exporting the Cq values of all six reference genes (18S, TBP, PGK1, TRFC, B2M and GAPDH) to the software geNorm version 3.5 where the gene expression normalization factor for each sample based on the geometric mean of the reference genes was calculated [38]. The reference genes were selected based on the gene expression stability measure M for a reference gene as the average pair wise variation V for that gene with all other tested reference genes. Based on these calculations PGK1, 18S, TBP, TFRC and B2M were selected as reference genes. Relative quantification was made using qbase^PLUS^ v.1.1 software [39]. The triplicates were allowed to differ by 0.5 Cq. The gene expression results were illustrated using GraphPad Prism 5.

### Microarray

RNA was isolated from cultures A and B during proliferation (long term (BG_0_) and after re-activation (AG_0_), quiescence (G_0_) and differentiation (DG_0_) and sent to Genotypic Inc. Bangalore, India where the microarray experiments were performed using Agilent whole human arrays 4×44 k (one-colour array). The experiments and the preliminary data analyses were done by Genotypic technologies, Bangalore, India (experimental protocols are available on http://www.agilent.com). The data was extracted by Agilent Feature extraction software and analysed using Genespring GX version 10.0 and Microsoft Excel. The samples were normalized using percentile shift normalization, which is a global normalization where the locations of all the spot intensities in an array are adjusted. The normalization took each column in an experiment independently, and computed the median of the expression values for this array, across all spots. Then the expression value of each entity was subtracted from the median value. The samples A and B were paired and differentially expressed genes in all 6 combinations (BG_0_/G_0_, G_0_/AG_0_, BG_0_/AG_0_, G_0_/Dif, AG_0_/Dif, BG_0_/Dif) were determined. The pathway analyses were performed using Biointerpreter (Genotypic). The data discussed in this study have been deposited in the NCBI Gene Expression Omnibus (GEO, http://www.ncbi.nlm.nih.gov/geo/) website and are accessible through GEO Series accession number GSE38769.

## Results

### G0 Arrest of Myoblasts

**Figure 1:**
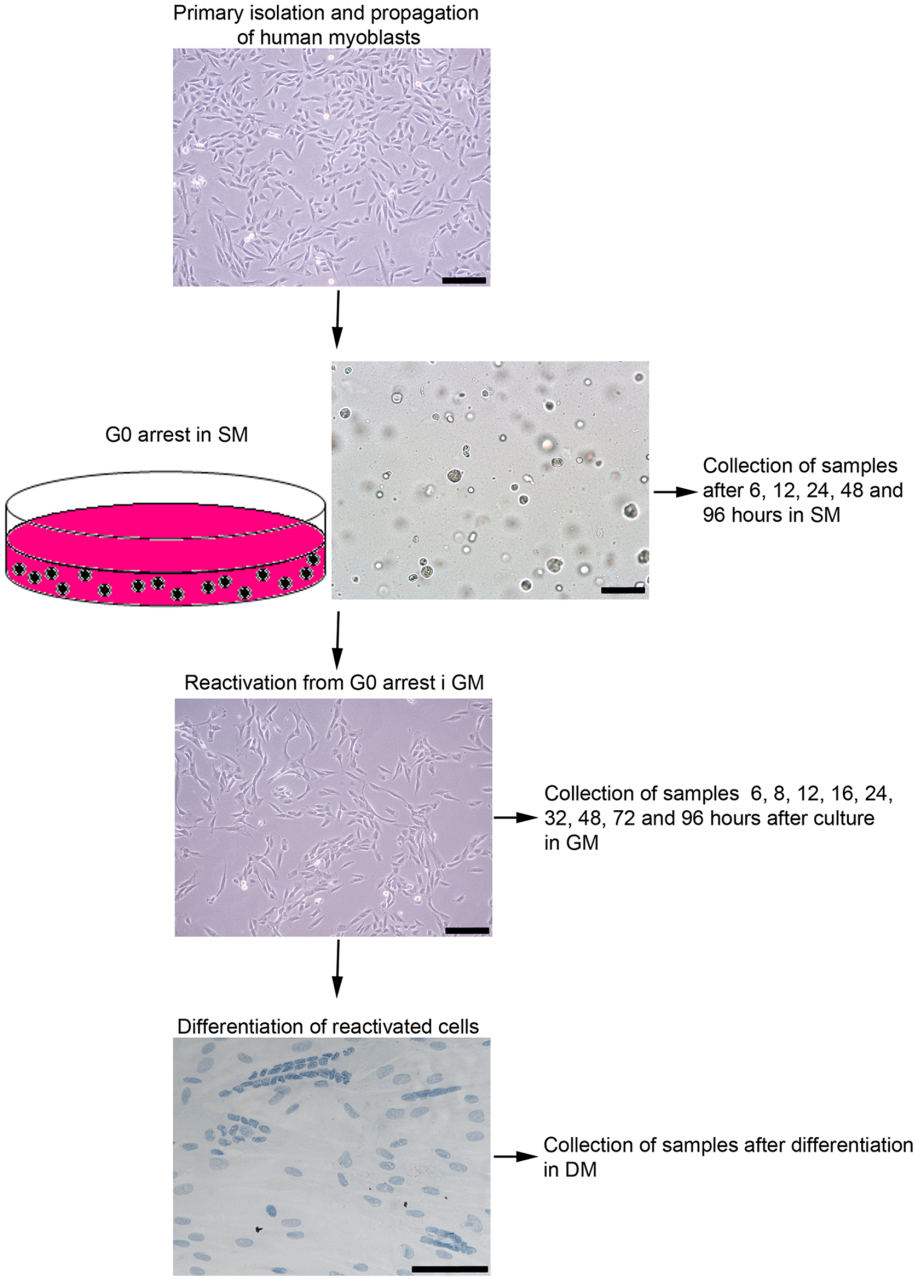
Scheme of the method for G_0_ arrest and reactivation of human myoblasts. Primary isolated myoblasts were expanded in GM, G_0_ arrested in SM, reactivated in GM and finally differentiated in DM. Samples for gene and protein expression studies were collected at different time points during G_0_ arrest, reactivation and after differentiation. In SM the cells rounded up and remained in this state for several days. Some of the G_0_ arrested cells formed doublets, due to lack of ability to drift apart after finishing the cell division they had started before transfer to SM. The cells did not start a new round of cell division after culture in SM. Scalebar: 100 µm.

Human myoblasts from 3 individuals, A, B and C, were isolated and cultured on ECM-coated dishes and pre-plated during every passage to remove contaminating fibroblasts. We have previously characterized the cell population isolated according to the described procedure and found that 95% of the cells were positive for the human myoblast marker NCAM [37], confirming that the isolation and culturing methods result in a highly purified satellite cell population. The isolated cells were cultured in growth medium (GM) and when sufficient amount of cells were obtained, they were transferred to suspension medium (SM) for growth arrest. Entry into quiescence was studied by collecting samples after 6, 12, 24, 48, 72, and 96 hours in SM. After 96 hours in non-adherent culture (SM96h) the cells were quiescent. These G_0_ cells were reactivated by transferring them to normal adherent culture conditions in growth medium (GM) and samples were collected for analysis after 5, 8, 9, 12, 16, 24, 32, 48, 72 and 96 hours in GM. Finally, cells were differentiated in differentiation medium (DM)). A scheme of the entire time course is shown in Figure 1.

**Figure 2:**
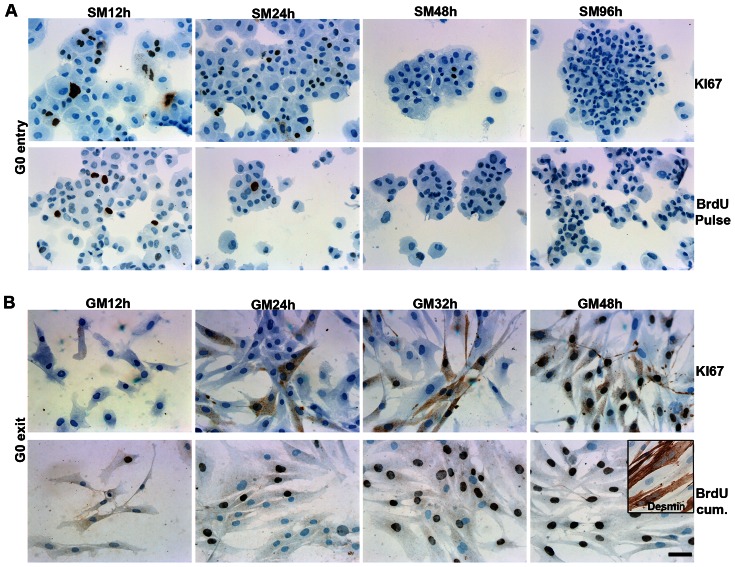
Expression of KI67 and incorporation of BrdU during G_0_ arrest and reactivation. (**A**) G_0_ arrested cells were cytospinned on coverglasses, which caused aggregation of the cells. KI67 and BrdU, which was incorporated as 1 h pulse, were detected by immunocytochemistry. Selected time points (12, 24, 48 and 96 hours) during culture in SM are shown, and a down regulation in the expression of KI67 and incorporation of BrdU was observed. At SM96h no KI67 or BrdU were detected, thus the cells have entered the G_0_ phase. (**B**) KI67 expression and BrdU incorporation is shown for selected time points after reactivation (GM12h, GM24h, GM32h and GM48h). KI67 expression was observed in a few cells at GM24h followed by a large up regulation during rest of the reactivation period and at GM48 most of cells were KI67 positive. During reactivation the cells were continuously exposed to BrdU, thus a cumulative BrdU incorporation is show. At GM12h only a few cells had incorporated BrdU but subsequently most of the cells became BrdU positive. Furthermore, the G_0_ arrested cells were able to fully differentiate and form desmin positive myofibers (B, insert). Thus, the cells were able to enter the cell cycle after G_0_ arrest. Scale bar: 50 µm.

The cell cycle status during growth arrest and reactivation was verified by assessment of KI67 expression (indicative of cells in G1/S/G2) and BrdU incorporation (quantify cells in S phase) (Figure 2). A decrease in expression of KI67 was observed in suspension cultures as early as SM6h and onwards and by SM96h, no KI67 expression was detected, indicating cell cycle arrest (Figure 2A). Consistent with arrest, by SM96h no DNA synthesis was detected by BrdU pulse exposure. Thus, culturing cells in non-adherent conditions for 96 hours resulted in cell cycle arrest, despite the presence of a full complement of serum (10% FBS). Thus, human myoblasts like mouse myoblasts are completely dependent on substrate attachment for normal proliferation.

To determine the kinetics of cell cycle entry, we analysed KI67 expression in arrested and reactivated cells. When the G_0_ arrested cells were reactivated by replating on adherent dishes, a few KI67 positive cells were observed starting at GM12h, but at GM24h a significant number of cells were expressing KI67, initially both in cytoplasm and in the nucleus (Figure 2B). By 48 hours in GM the majority of the cells were positive for KI67.

To assess the fraction of arrested cells capable of re-entering the cell cycle, cells were continuously exposed to BrdU during the entire period of reactivation and the cumulative incorporation detected; selected time points for BrdU incorporation are shown in Figure 2B. As with KI67 staining, few BrdU positive cells were observed at 12 hours after replating and this number increased dramatically, peaking at 32 hours after reactivation. The reactivated cells were also induced to differentiate and were able to form multinucleated myofibers expressing desmin (Figure 2B, insert). Furthermore, the G_0_ arrested cells were able to enter and exit a second round of G_0_ arrest (results not shown).

**Figure 3:**
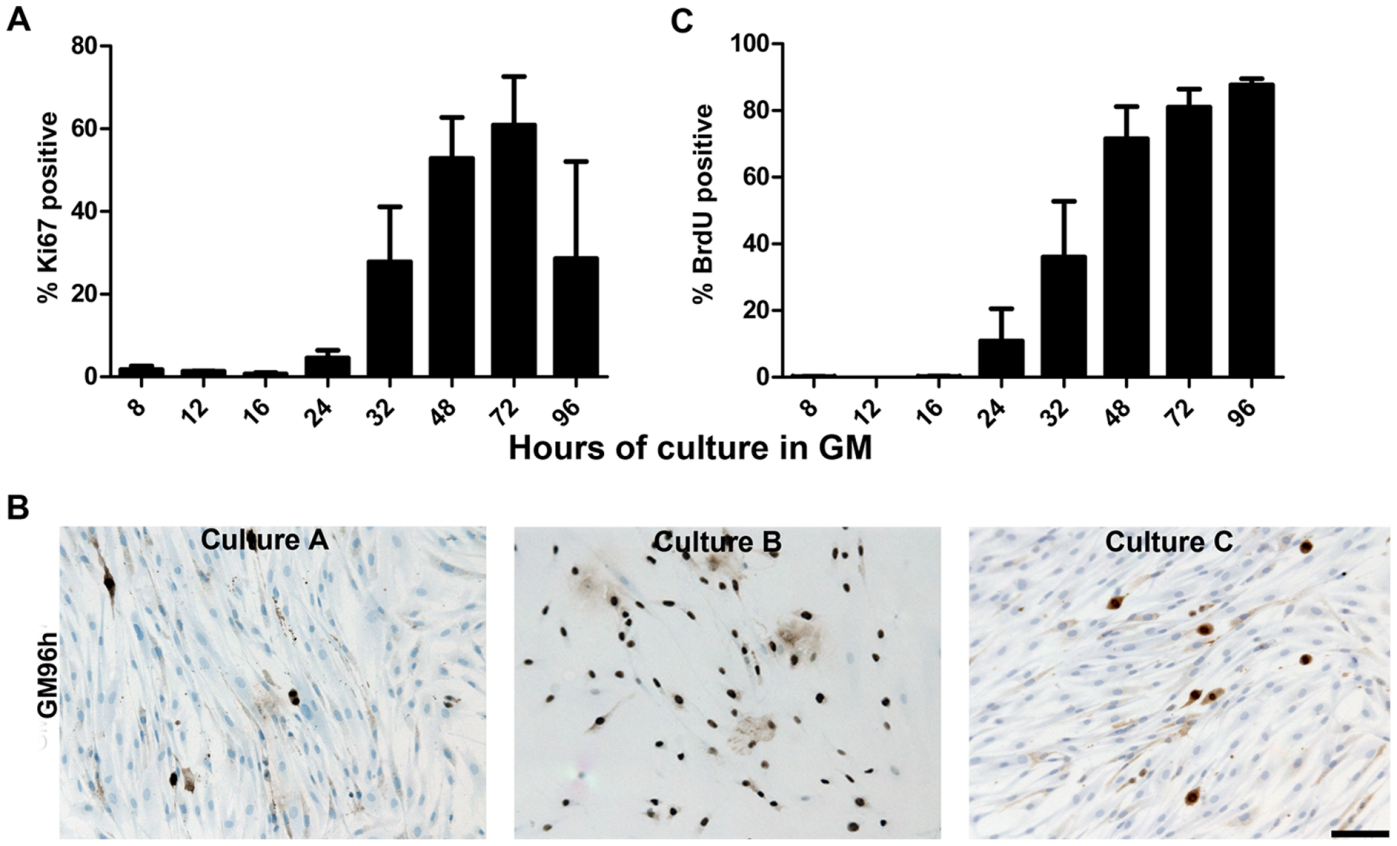
The proliferation potential of myoblasts when activated from G_0_. (**A**) The fraction of Ki67 positive cells in the three cell cultures at different time points during activation is shown. From GM24h to GM72h a major increase in Ki67 fraction was observed followed by a down regulation at GM96h. The large standard errors are due to differences in growth rate. Cell culture A and C had a higher growth rate and became confluent by GM96h with only few cells expressing Ki67, while culture B was still sub confluent and had a large fraction of cells still expressing Ki67 (**B**). (**C**) The fraction of BrdU positive cells was determined after reactivation in GM. A large increase in BrdU incorporation was observed from GM24h and by GM96h 87.7% (±1.8 SEM) of the cells were able to incorporate BrdU and thus reenter the cell cycle. Scale bar: 100 µm.

The fraction of KI67 positive cells, during reactivation from G_0_ was determined for the three cell cultures and mean values are shown in Figure 3A. A major increase in the KI67**^+^** fraction was observed at GM32h which increased up to GM72h, but decreased at GM96h The large standard errors are due to individual variations in proliferation rate among the three cell isolates from different subjects (Figure 3C). Due to a higher proliferation rate, cultures A and C reached confluence at GM96h with only few cells expressing Ki67. Culture B had not yet reached confluence at GM96h and cells were still in a proliferative phase, consistent with persistent Ki67 expression. Despite the variation in growth rate of myoblasts derived from different individuals, the overall trend was very similar.

The fraction of BrdU positive cells revealed that an increasing number of cells entered S-phase during culture in GM starting at GM24 (Figure 3B). By 96 h in GM, 87.7% (±1.8 SEM) of the cells were BrdU positive in a cumulative exposure set up. Thus, the majority of G_0_ arrested cells were able to re-enter the cell cycle when replated in adherent conditions in GM.

### Regulation of Cell Cycle during Go Entrance, Exit, and Differentiation

To determine the expression of cell cycle and myogenic genes in the three cultures A, B and C we used real time reverse transcription PCR (qRT-PCR). RNA was isolated at six time points during entry into G_0_ arrest (SM), nine time points during reactivation (GM) and finally after differentiation (DM). The results for the individual genes are shown as fold changes where the lowest normalized Cq value for each gene was set to 1. This allowed us to detect differences in expression level between the cultures in addition to the temporal development.

**Figure 4:**
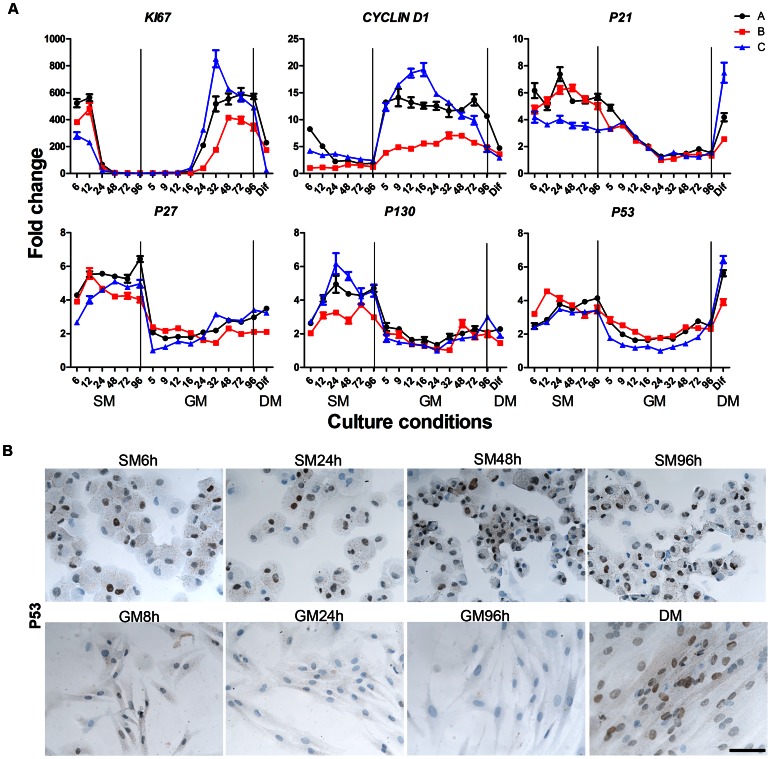
Expression levels of cell cycle related genes during G_0_ entrance (SM), exit (GM) and differentiation (DM). Cell cultures A, B and C were cultured in suspension-, growth-, and differentiation medium, and qRT-PCR was performed at different time point during the study (A). *KI67* expression was highly down regulated during G_0_ arrest between SM12h and SM24h and after reactivation we observed a major up regulation between GM16-GM48h followed by a large down regulation after differentiation. *CYCLIN D1* was expressed at low levels during G_0_ arrest but after activation a large up regulation was detected already after 5 hours. In the late period of reactivation the expression was declining with further down regulation after differentiation. Expression levels of *P21*, *P27*, *P130* and *P53* were high during G_0_ arrest, but after activation in GM the expression levels dropped followed by a small up regulation in the late GM samples. Furthermore, P21 and P53 were markedly up regulated after differentiation. The protein expression of P53 during G_0_ arrest, reactivation and after differentiation is shown in (B). High levels of P53 were observed during G_0_ arrest, followed by down regulation after activation. After differentiation the expression of P53 was again up regulated. Thus, the gene expression correlated with the protein expression. Scale bar: 100 µm.

We found a rapid down regulation of *KI67* occurring between 12 h and 24 h after culture in suspension medium (SM12-24h), a clear indication that the cells were exiting cell cycle (Figure 4A). Upon reactivation in GM, a tremendous *KI67* up regulation from GM24-32h, was observed with a 400–800 fold difference compared to late SM samples, indicating a synchronous activation of the cell cycle. As expected, KI67 expression was down regulated when cells were induced to differentiate (irreversible cell cycle exit).

During the last period of G_0_ entry (from SM24h onwards), cell cycle regulators (*CYCLIN D1*, *P21, P27, P130* and *P53),* appeared to reach a stable expression pattern, and thus a steady state in cell cycle arrest was apparent. By contrast, even by five hours after reactivation, marked changes in gene expression were observed. *KI67* was up regulated at GM24h, following altered expression of *CYCLIN D1*, *P21, P27, P130* and *P5*3.

*CYCLIN D1* is a regulator of G_1_/S phase transition and is required throughout the G1 phase, but declines as cells enter the S phase [Bibr pone.0064067-Stacey1] [40,41]. We found that *CYCLIN D1* expression was low in G_0_ arrested cells, followed by a large up regulation at 5 hours after reactivation, consistent with entry into G1. In all three cell cultures, *CYCLIN D1* expression decreased again during progression through G_1_ to S phase. A further decrease was observed in differentiated cultures. These results support the interpretation of a reversible cell cycle arrest or quiescence in SM followed by a synchronous reactivation during replating in GM.

Regulation of quiescence and activation was further studied by the expression profiles of negative regulators of the cells cycle-P21, P27, P53 and P130. P53, a tumor suppressor gene, is a major factor controlling cell proliferation and is known to play a role in cell cycle arrest. Activation of P53 is driven by various stress signals, including loss of normal cell contact [Bibr pone.0064067-Farnebo1] [42,43]. P21, a cyclin-dependent kinase inhibitor, functions as a downstream mediator of P53 [44–46] and accumulation of P53 triggers activation of P21 leading to cell cycle arrest or apoptosis [43].

Consistent with their roles as negative regulators, *P21, P27, P130* and *P53* were all expressed at higher levels during G_0_ arrest compared to reactivation. *P27* and *P130* were rapidly down regulated within the first 5 h after activation, whereas *P21* and *P53* were down regulated with slower kinetics, with p53 reaching the lowest levels at GM12-16, followed by p21 at GM24. This is consistent with P21 being downstream to P53. Furthermore, P21 and P53 were up regulated during differentiation. P53 protein levels were evaluated by immunostaining studies; almost all cells became P53 positive during G_0_ arrest (Figure 4B). A down regulation of P53 was observed after reactivation and after GM48h no P53 expression was observed in the cell nuclei, however after differentiation the expression was again up regulated. Thus, the protein expression of P53 was consistent with mRNA expression.

P27 has in previous studies been associated with quiescence [32,47,48] and our studies support those findings. P130 is a regulator of cell growth and differentiation and a candidate tumor suppressor. It has been shown that the formation of a complex between P130 and E2F, regulated by phosphorylation, is unique to cells in a quiescent, G_0_ state [Bibr pone.0064067-Hansen1] [49,50]. Expression of *P130* at a higher level in G_0_ arrested cells compared to proliferating cells suggests that the components of the complex are also present in quiescent human myoblasts.

### Expression Pattern of PAX Genes and MRFs during G_0_ Entrance, Exit and Differentiation

**Figure 5:**
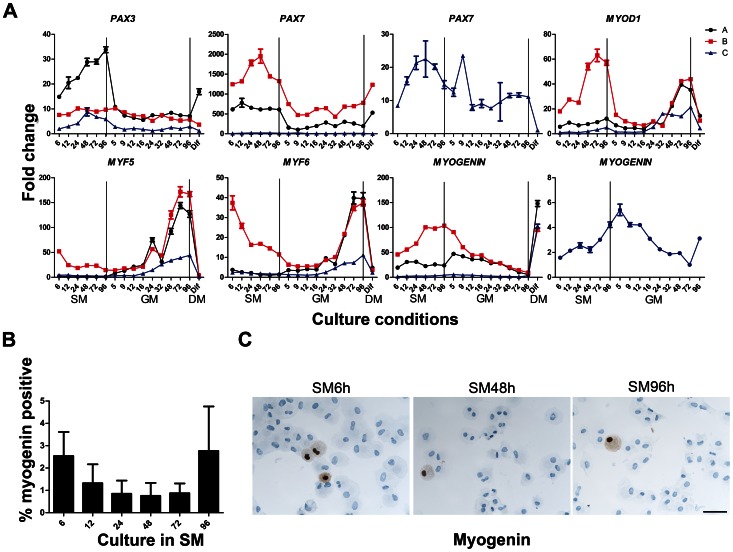
Expression of *PAX* genes and MRFs during G_0_ entrance (SM), exit (GM) and differentiation (DM). (A) *PAX3* and *PAX7* expression levels were high in G_0_ arrested samples but became down regulated when cells were reactivated. Expression of MyoD1 seemed to increase during G_0_ arrest followed by a drop in expression during early reactivation. In the later phase of reactivation the expression was up regulated and finally down regulation after differentiation. The markers *MYF5* and *MYF6* expressions were relatively stable during G_0_ arrest for cultures A and C but after activation the expression of all three genes became up regulated followed by a large down regulation after differentiation in all three cultures. In cultures B and C, *MYOGENIN* expression was approx 2-fold up regulated during G_0_ arrest, but after reactivation all three cultures had a drop in *MYOGENIN* expression followed by up regulation after differentiation. (B, C) The protein expression of MYOGENIN during G_0_ entrance was studied by immunocytochemistry. After 6 h in SM only 2.5% (±1.1 SEM) of the cells were MYOGENIN positive and the fraction decreased further during culture in SM with a tendency for an increase up to 2.8% (±2.0 SEM). Thus, MYOGENIN protein expression did not seem to correlate completely with gene expression. Scale bar: 100 µm.

*PAX3* and *PAX7* are homologous genes important for specification of myogenic stem cells and known to be expressed by quiescent satellite cells [51–53]. In our model of quiescence, expression of *PAX3* increased during G_0_ arrest in cell cultures A and C, while expression in B did not change noticeably (Figure 5A). In GM5h *PAX3* was down regulated in cultures A and C compared to SM72-96h, followed by a lower, almost constant expression in the rest of the reactivation period. Expression of *PAX3* in Culture B did not vary much and showed only a minor tendency for down regulation in the late reactivated samples compared to G_0_ arrested samples. *PAX7* expression was up regulated during G_0_ arrest in B and C, while the expression in A seemed almost constant (Figure 5A). After reactivation *PAX7* expression was down regulated already at GM5h in cultures A and B followed by a similar, lower wave shaped expression. The overall *PAX7* expression during reactivation in culture C also seemed lower compared to G_0_ arrested cells. During differentiation *PAX7* was down regulated in culture C but up regulated in cultures A and B. Cells in culture C, originating from m. gluteus maximus, had low levels of *PAX7* compared to the other two cultures but the pattern of expression tended to be similar mostly to culture B. Noticeably, cell culture B which had the least variation in *PAX3* expression had the highest variation and expression level of *PAX7*. In summary the *PAX* genes in myoblasts demonstrated inter individual differences.

Myogenic Regulatory Factors (MRFs) include MYOD and MYF5, involved in early activation of satellite cells, and MYF6 and MYOGENIN, involved in differentiation of myoblasts [Bibr pone.0064067-Buckingham1] [Bibr pone.0064067-Olson1] [54–56].

*MYF5* levels were low in all three cultures in SM and early GM but starting from 16–24 hours in GM, a 50–150 fold up regulation was observed which persisted during late GM (Figure 5A). *MYF5* expression was markedly down regulated after differentiation.

We found increased levels of *MYOD1* in SM96h compared to SM6h (Figure 5A). After reactivation the expression level dropped and when cells began to proliferate, an increase was again observed, consistent with the G1 induction of MyoD observed in mouse satellite cells. Notably, culture B had high levels of *MYOD1* expression during quiescence. In all 3 cultures differentiation resulted in a 20–40 fold decrease.

*MYF6* was low expressed in cultures A and C during G_0_ arrest and in GM5-16h. In culture B, there was a large down regulation throughout G_0_ arrest, which also reached a minimum at GM5-16h, after which all 3 cultures had a substantial up regulation followed by an equal substantial down regulation after differentiation. Thus, *MYF6* expression seemed to follow *MYF5* expression.

*MYOGENIN* expression increased during G_0_ arrest in cultures B and C, whereas in culture A it remained almost constant. All 3 cultures had a marked drop in *MYOGENIN* after reactivation in GM consistent with the absence of differentiation in these synchronized proliferating cultures. However, an expected large up regulation of Myogenin was seen when cells were transferred to DM.

**Figure 6:**
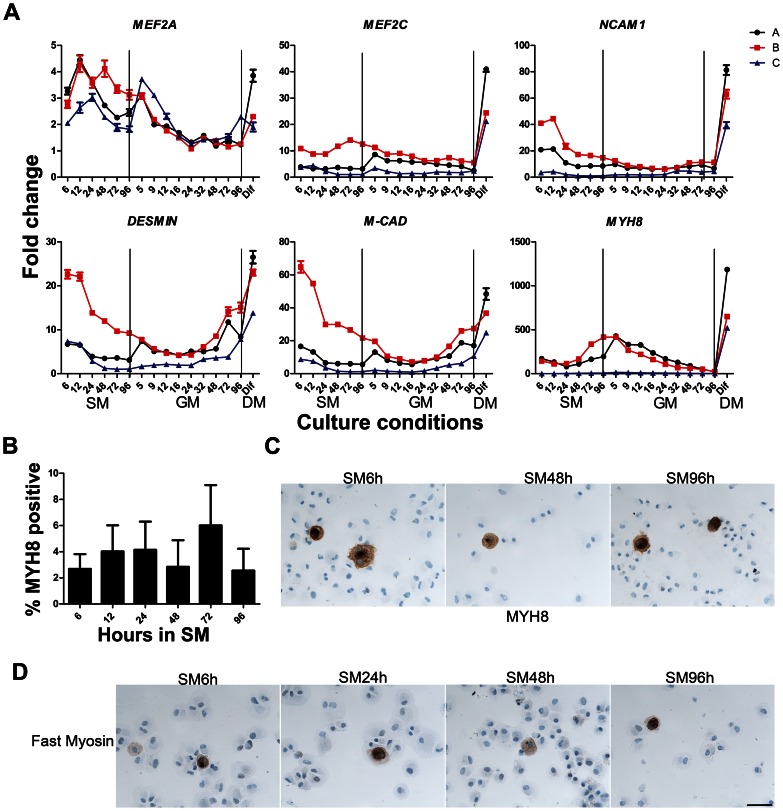
Gene expression of early and late markers of myogenesis during G_0_ entrance, exit and differentiation. (A) *MEF2A* and *MEF2C* were all expressed throughout G_0_ arrest and re-activation, with peaks seen in SM and early GM samples followed by up regulation after differentiation. *NCAM*, *DESMIN* and *M-CAD* expressions were high in the early SM samples followed by down regulation and finally up regulation in the late GM samples and after differentiation. *MYH8* was up regulated during G_0_ arrest but became down regulated in the reactivated samples after GM5h and largely up regulated after differentiation. (B,C) Protein expression of MYH8 was studied by immunocytochemistry and the fractions of MYH8 positive cells were determined during G_0_ arrest. MYH8 seemed to be present in a small portion of the cells throughout G_0_ entrance, however no correlation between gene and protein expression was observed. (D) Immunostainings of Fast Myosin during G_0_ entrance showed a few positive cells, an expression similar to MYH8. Scale bar: 100 µm.

The protein expression of MYOGENIN during G_0_ entry was also studied and the fraction of positive cells was determined for each of the six time point (Figure 5B). After 6 h in SM only 2.5% (±1.1 SEM) of the cells were MYOGENIN positive and the fraction decreased further during culture in SM with a tendency for an increase up to 2.8% (±2.0 SEM). Thus, MYOGENIN protein expression did not seem to correlate with gene expression, suggesting that although transcript levels may have increased in some non-adherent cells, the protein was not made and therefore, the quiescent cells resisted differentiation. Figure 6C illustrates the protein expression of MYOGENIN at selected time points during G_0_ arrest.

### Expression of Myogenesis-related Genes

*MEF2A* and *MEF2C* are among the early markers during muscle development. They are not sufficient to induce myogenesis [Bibr pone.0064067-Dodou1] [Bibr pone.0064067-Black1] [Bibr pone.0064067-Molkentin1] [57–60], but required to stabilize and enhance differentiation [Bibr pone.0064067-Ornatsky1] 60,61. During G_0_ entry we observed a small peak of *MEF2A* in early SM samples followed by a small down regulation in the late SM samples (Figure 6A). Culture C had a small peak in GM5h, but otherwise all three cultures slowly down regulated *MEF2A* and the expression reached a steady level in all samples from GM24h. *MEF2C* expression pattern was similar in A and C, with low levels in late SM samples followed by a small up regulation in GM5h after which the expression again dropped slowly. In culture B *MEF2C* expression was induced from SM24-96h but after reactivation the expression level dropped slowly. *MEF2A* and *MEF2C* levels were highly induced during differentiation.

The expression pattern of *NCAM1*, *DESMIN* and *M-CADHERIN (M-CAD)* were almost similar, with high expression levels in the early SM samples, down regulation in late SM and early GM (Figure 6A). *DESMIN* and *M-CAD* expressions increased approx 32 hours after reactivation and *NCAM1* also seemed to have a small tendency to increase around the same time. All three genes were up regulated after differentiation. *MYH8* mRNA levels increased during G_0_ arrest, followed by a drop in expression during the entire reactivated period, but after differentiation we observed a large up regulation (Figure 6A). The cells originating from gluteus maximus had lower levels of *MYH8*, but similar expression profile to the other cultures.

The protein expressions of neonatal and adult isoforms of myosin heavy chain for culture A, B and C during G_0_ entrance was studied by immunocytochemistry. The mean fractions of MYH8 (neonatal isoform) positive cells were determined (Figure 6B). MYH8 protein seemed to be present in a small portion of the cells during G_0_ entry and after 96h in SM only 2.6% (SEM ±1.7) of the cells were MYH8 positive. However the MYH8 fraction varied during G_0_ arrest and low protein expression did not reflect the induced mRNA levels, again suggesting that lack of overt differentiation. Immuno-detection of MYH8 at selected time points during G_0_ arrest is shown in Figure 6C. The expression of the adult isoform of myosin heavy chain, Fast Myosin, at selected time points during G_0_ arrest was determined (Figure 6D) and in line with the expression of MYH8, only a few cells were positive for Fast Myosin.

### Expression of cMET, FGFR1, and FGF2 and SGCA

**Figure 7:**
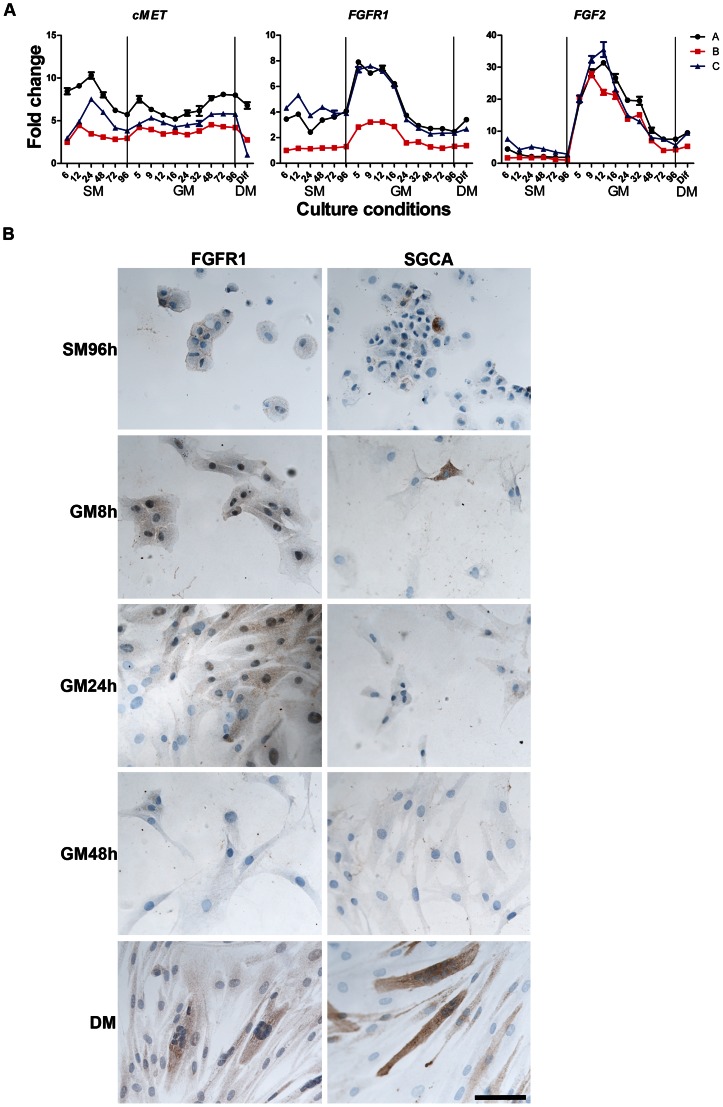
Gene expression of cMET, FGFR1 and FGF2 during G_0_ entrance, exit and differentiation. *cMET* had a wave shaped expression during G_0_ entrance and exit. *FGFR1* and its ligand *FGF2* were highly up regulated in the early phase of reactivation and down regulated in late phase and after differentiation. Immunocytochemical analyses of FGFR1 correlated with gene expression, with low levels of FGFR1 during G_0_ arrest and up regulation immediate after reactivation followed by down regulation at GM48h (B). Only a few SGCA positive cells were observed at G_0_ arrest (SM96h) and after replating (GM8h), however after differentiation both FGFR1 and SGCA were upregulated in myofibers. Scale bar: 100 µm.

*C-MET,* the receptor for HGF or scatter factor is expressed in both resting and proliferating myoblasts and is involved in satellite cell activation [Bibr pone.0064067-Cornelison1] [30,62]. We found expression of *C-MET* mRNA in cells during G_0_ arrest and reactivation (Figure 7) and the expression pattern for all three cultures showed two distinct peaks at SM12-24h and GM5-9h, and a tendency for down regulation after differentiation. This pattern is consistent with a role for c-met in activation.

The growth factor *FGF2* is also important for stimulating satellite cell proliferation [63,64] and exerts this function through the receptor *FGFR1*. Our results showed constant levels of gene expression for *FGFR1* and *FGF2* throughout SM, but immediately after reactivation, *FGFR1* levels doubled and around GM24-32h FGFR1 was again down regulated and reached almost same expression levels as in SM. Within 5 hours of reactivation *FGF2* levels increased up to 20-fold at GM5h and 30-fold at GM9h. Down regulation of *FGF2* during reactivation occurred slowly, starting after GM9-12h and the expression level seemed to have reached a stable level in late GM with no significant changes after differentiation. The peak in *FGFR1* and *FGF2* appeared before the increase in *KI67* expression, and thus ahead of proliferation. These results are consistent with a role for *FGFR1* and *FGF2* in activating human satellite cells.

It has been reported that FGFR1 interacts with SGCA in regulating commitment of murine and human myoblasts (ref: Cassano M 2011), and this notion was tested in our model by studying the expression of FGFR1 and SGCA using immunocytochemistry (Figure 7B). A low level of FGFR1 was expressed in G_0_ arrested cells, with up regulation immediately after reactivation lasting until GM48. Only a few cells were SGCA positive at SM96h and immediately after replating (GM8h). However, FGFR1 and SGCA were up regulated in differentiated myofibers, indicating and interaction during differentiation.

Taken together, the results of analysis of candidate cell cycle and myogenic genes indicate that human myoblasts can enter quiescence in culture, can be viably maintained in this state, which can be used to generate synchronously proliferating cultures.

### A Global Profile of Quiescent and Activated Human Myoblasts

In order to reveal global differences between asynchronously proliferating, quiescent, reactivated and differentiated human muscle cells, we used transcriptome profiling. A microarray study including samples A and B was designed in order to detect the differences in global gene expression of myoblasts during four different culture conditions, two proliferating and two non-proliferating. (Sample C was omitted as it had a distinct muscle origin). Myoblasts were maintained as long term asynchronously proliferating cultures (designated as BG_0_, before G_0_) followed by G_0_ arrest for 96 hours in suspension culture (designated as G_0_), synchronously reactivated (designated as AG_0_, after G_0_) and finally they were differentiated (designated as Dif). The samples BG_0_ and AG_0_ were harvested when cells reached 70–80% confluence.

Total RNA was isolated from all 4 samples (2 independent cultures, A and B each), labeled and used to interrogate Affymetrix human arrays. The raw data from gene arrays were normalized, sample A and B were paired and the mean expression value used for further analyses. The four samples were compared to each other and the number of genes with ≥2-fold differential expression is shown in table 1.

**Table 1:** Comparison of gene expression in BG_0_, G_0_, AG_0_ and Dif.

	Number of genes≥2-fold difference	Highexpression	Lowexpression
**BG_0_ vs G_0_**	5032	50,1%	49,9%
**G_0_ vs AG_0_**	6404	50,0%	50,0%
**BG_0_ vs AG_0_**	4598	52,4%	47,6%
**G_0_ vs Dif**	4812	42,8%	57,2%
**AG_0_ vs Dif**	5448	43,3%	56,7%
**BG_0_ vs Dif**	5669	46,6%	53,4%

Number of genes with ≥2-fold differential expression for the six comparisons are shown and the persentage of high and low expressed genes are calculated.

G_0_ compared to AG_0_ (G_0_/AG_0_) had 6404 differentially expressed genes, thus the biggest difference in gene expression was observed in the transition from G_0_ to reactivation. Noticeably, the G_0_ arrested state did not merely reflect a shutdown of gene expression, since equivalent numbers of the genes showed high and low expression in G_0_/AG_0_. G_0_ compared to differentiation had 4812 differentially expressed genes, 42.8% with higher expression in G_0_ indicating that different mechanisms are active in the two non-proliferative states.

The two samples with proliferating cells before and after G_0_ (BG_0_ and AG_0_) might be expected to be similar, but showed 4598 differentially expressed genes and were thus considerably different.

In order to dissect the transcriptional pathways underlying the observed differential expression, pathway analyses was performed with the top 1000 differentially expressed genes in each of the three comparisons G_0_/AG_0_, G_0_/Dif and BG_0_/AG. The results of highly expressed genes in a selection of signaling pathways involved in the respective comparisons with a p-value <0.05 are shown in Table 2. In the transition from G_0_ to reactivation (G_0_/AG_0_), genes in Wnt and calcium signaling pathways were highly expressed, indicating that these pathways may play significant roles. Furthermore, cell adhesion molecules were differentially expressed which might reflect the transition from suspension to adherent cultures during quiescence and reactivation.

**Table 2:** Pathway analyses were made with the top 1000 differentially expressed genes in each of the three comparisons G0/AG0, G0/Dif and BG0/AG.

High expressed genes in G_0_/AG_0_					
Pathways		No. of genes	Gene list	P value	
Cytokine-cytokine receptorinteraction (269)		10	*IL7R, TNFNRSF11B, LEP, KIT, cMET, CCL5, CX3CR1, CD70, CXCL12, INHBB*	1.00E-09	
Regulation of actincytoskeleton (230)		14	*DIAPH3, FGFR2, ACTC1, FGF12, FGF1, ACTA2, ACTG2, BDKRB1, MYLC2PL, MYLK, ARGHEF4, ITGB2, SCIN, ACTN4*	1.00E-09	
Focal adhesion (217)		10	*THBS1, RELN, COMP, LAMA3, PGF, MYLC2PL, MYLK, cMET, CCND1, ACTN4*	0.0005	
Wnt signaling pathway (158)		8	*DKK1, PLCB4, TCF7L1, CCND1, FZD10, DKK2, WNT5B, TCF7L2*	0.0053	
Cell adhesion molecules,CAMs (145)		7	*ALCAM, PTPRF, NCAM2, ITGB2, WWC2, NRXN3, CDH4*	0.006	
Calcium signaling pathway (196)		12	*SLC8A1, PDE1C, HTR2A, BDKRB1, MYLK, ADRB2, ADRA1B, PLCE1, OXTR, BMST1, ADCY4, PLCB4*	0.007	
ECM-receptor interaction (95)		5	*HMMR, THBS1, RELN, FNDC1, LAMA3*	0.0364	
**High expressed genes in BG_0_/AG_0_**					
**Pathways**	**No. of genes**		**Gene list**		**P value**
Regulation of actincytoskeleton (230)	10		*DIAPH3, ACTC1, FGF1, ACTG2, FGFR2, MYLC2PL, SCIN, IGGA10, ACTA2, PPP1R12B*		0.0001
Wnt signaling pathway (158)	6		*PRKY, DKK1, PLCB4, TCF7, AXIN2, SFRP1*		0.0007
Cell signaling pathway (196)	9		*OXTR, PRKY, PLCB4, ADRA1B, SLC8A1, PTGER3, ATP2B4, BST1, CAMK4*		0.0005
Focal adhesion (217)	13		*FLT1, CAV3, HGF, TPEN, PGF, MYLC2PL, CAV1, THBS1, ITGA10, FLNC, AKT2, LAMA3, CAV2*		0.0021
Cell communication (146)	7		*KRT34, THBS1, LMNB1, KRT19, LAMA3, KRT83, GJA3*		0.0036
Cell adhesion molecules,CAMs (145)	7		*HLA-DMB, NEGR1, ALCAM, HLA-DRA, WWC2, NACM2, MADCAM1*		0.0039
ECM-receptor interaction (95)	4		*HMMR, THBS1, ITGA10, LAMA3*		0.0128
**High expressed genes in G_0_/Dif**					
**Pathways**	**No. of genes**		**Gene list**	**P value**	
Cell cycle (123)	28		*E2F2, CCNA2, CDC45L, PKMYT1, CDC2, BUB1, BUB1B, CDC25C, CCNB2, PLK1, CDC20, E2F1, CCNB1, PTTG2,* *ESPL1, PTTG1, ORC1L, CDC6, ORC6L, CDC25A, SMC1B, TFDP1, RBL1, CCNE2, MCM5, CDK2 GADD45B, MCM2*	1.00E-09	
MAPK signaling pathway (276)	13		*FGFR3, BDNF, NTF3, GFGP, HSPA2, FGFR2, FGF1, FGF2, FLNB, DUSP2, GADD45B, NR4A1, FLNC*	1.00E-09	
Regulation of actincytoskeleton (230)	15		*FGFR3, DIAPH3, BDKRB1, MYLC2PL, FGF9, FGFR2, ITGB8, FGF1, FGF2, ITGB2, F2R, ACTA2, TIAM2,* *PIP5K1A, ITGB7*	1.00E-09	
Cytokine-cytokine receptorinteraction (269)	11		*IL7R, INHBB, CXCL1, CXCL5, CXCL6, IL6, CD70, CCL5, TNFRSF11B, CSF3, IL21R*	1.00E-09	
Focal adhesion (217)	9		*MYLC2PL, THBS1, ITGB8, BIRC3, CAV2, FLNB, ITGB7, TNXB, FLNC*	0.0001	
Cell adhesion molecules,CAMs (145)	6		*ITGB2, ITGB7, ITGB8, L1CAM, NRXN3, ALCAM*	0.0014	
Calcium signaling pathway (196)	14		*ADRB2, BDKRB1, PDE1C, SLC81A, ADCY4, BST1, ATP2B4, OXTR, ADRA1B, F2R, P2RX5, HTR2A, TRHR, P2RX1*	0.0096	

Table 2 shows high expressed genes in a selection of signaling pathways involved in the respective comparisons with a p-value <0.05. Numbers within the brackets specifies the number of genes included in the pathway.

In G_0_/Dif, 28 of 123 genes involved in cell cycle were differentially expressed, indicating that the reversible and irreversible cell cycle arrest observed in the two samples were regulated by different mechanisms. Again genes in Wnt and calcium signaling pathways, cell adhesion molecules and genes in the regulation of actin cytoskeleton were differentially expressed.

In the comparison of asynchronous and synchronized proliferation (BG_0_/AG_0_) many genes in e.g. Wnt, Calcium, P53, Notch and TGFβ signaling pathways were differentially expressed, supporting the notion that these cultures of proliferating cells are in distinct states.

## Discussion

Skeletal muscle regeneration has been extensively studied with a focus on the regulation of satellite cell proliferation and differentiation [Bibr pone.0064067-Charge1] [Bibr pone.0064067-Holterman1] [5,65,66]. The programs that control the cell cycle and differentiation are coordinated to ensure the correct balance of stem cells and differentiated cells in regenerating tissue, but the mechanisms that direct the cells into reversible arrest and induce their initial activation are poorly understood [10]. Earlier studies have shown that suspension culture of mouse fibroblasts and C2C12 myoblasts results in cell cycle arrest in the G_0_ phase [Bibr pone.0064067-Milasincic1] [Bibr pone.0064067-Dhawan3] [33,67,68]. We have extended these studies to develop an in vitro model for physiologically relevant human myoblasts. Our results show that primary isolated human myoblasts from three independent human samples and two different muscle sources can be arrested in G_0_. We further characterize this model using transcriptome profiling and propose synchronized human myoblasts as a useful tool to elucidate the mechanisms controlling G_0_ arrest and early activation.

### The G_0_ Model

Cell cycle arrest of human myoblasts was observed after culturing the cells in suspension in a high viscosity medium containing 2% methyl cellulose for 96 h. G_0_ was confirmed by lack of Ki67 expression and DNA synthesis. When G_0_ arrested cells were reactivated by restoration of substrate contacts, 87.7% (±1.8 SEM) of the cells re-entered cell cycle by 96 h, verifying that the suspension-induced arrest was indeed reversible. This temporal pattern in vitro resembles that of the process of regeneration in vivo where myoblast replication and fusion are essentially completed by 5–7 days after experimental injury in animals [69,70].

Furthermore, a total shutdown of *KI67* and repressed levels of *CYCLIN D1* in suspended myoblasts followed by a dramatic up regulation during replating, supports the characterization of cells in SM as quiescent, but become rapidly and synchronously activated when exposed to substrate attachment. High expression levels of *P53*, *P21*, *P27,* and *P130* have been reported to correlate with cell cycle arrest [32,42,43,47–50]. Our results showing up regulation of these genes in SM further supports the notion that cells in SM enter G_0_. The finding that the initial changes in *P53*, *P21*, *P27* and *P130* during reactivation occurred before the major increase in *KI67* also fits with the expected sequence. Also, for KI67 we observed a steep increase in gene expression preceding the substantial induction of protein expression.

These basic experiments demonstrated that like mouse myobasts, human myoblasts were able to enter and exit cell cycle arrest depending on a simple alteration of culture conditions, i.e. prevention of cell attachment, without alterations in the growth factor/serum concentration. Other methods for G_0_ arresting cells have been described. Inhibition of acto-myosin contractility using the myosin inhibitors, ML7 and BDM, induces reversible G_0_ arrest in C2C12 myoblasts [32] and induction of GAS1 and FOXO3a expression resulted in reduced proliferation in mouse fibroblasts and human colorectal carcinoma cells, respectively [Bibr pone.0064067-Kops1] [Bibr pone.0064067-Evdokiou1] [71–73]. However, our suspension model for G_0_ arrest is simple and effective and does not involve either pharmacological agents or over-expression of genes, which could have unintended effects. Furthermore, the synchronized activation observed in our model makes it possible to detect changes in gene expression that would otherwise be masked in asynchronously proliferating populations.

The experiments also demonstrated that significant changes in gene expression occurred within a few hours after activation from G_0_ arrest, indicating that isolation of satellite cells from their tissue niche will result in activated cells, and that obtaining truly quiescent cells would be difficult if not impossible even with short isolation protocols.

### Expression of PAX, MRFs and Myogenesis Related Genes during G_0_ Arrest

Quiescence is characterized not only by absence of DNA synthesis but also suppression of differentiation, which permits resting cells to persist in a state from which they can be activated. The G_0_ culture model was further characterized by analysis of key myogenic regulatory factors that have been reported to play a role in satellite cells. We studied expression of selected markers in human myoblasts during G_0_ arrest, reactivation and differentiation. Induction of *PAX3* and *PAX7* and suppression of *MYF5* and *MYF6* in SM supported the conclusion that our myoblast cultures had entered an undifferentiated quiescent state 51–56. Considering the almost constant level of *PAX3* in sample B during SM and GM, one might question the degree of quiescence for this particular sample. However, the up regulation of *PAX7* in SM for even this sample, combined with the down regulation of *NCAM*, *DESMIN* and *M-CAD*, genes normally active in the late phases of myogenesis, suggests that the cells are in a non-differentiated arrested state.

Although early and late myogenic mRNAs (*MYOD1*, *MYOGENIN,* and *MYH8)* were up regulated during G_0_ arrest, only a small percentage of the G_0_ arrested cells showed expression of myogenin, myh8 and fast myosin protein expression (Figure 5B and 6B), indicating that translational control mechanisms prevented overt differentiation except for this minor proportion of cells.

It has been suggested that MyoD has an inhibitory effect on cell cycle independent of its myogenic function [74] which might be the case in G_0_ arrested human myoblasts, but this hypothesis is not consistent with the idea that absence of MyoD in G_0_ may be necessary for arrest to be reversible [75].

The finding that *MYOD1*, *MYOGENIN* and *MYH8* were up regulated during G_0_ arrest calls for further analyses. The explanation for these findings may be found in the conditions prior to G_0_ arrest. Proliferating cells are a mixture of cells in different phases of cell cycle and some cells may even have entered a differentiation pathway. Thus, sorting out these cells might provide a more homogenous population for future studies**.**

### Activation and Differentiation of G_0_ Arrested Human Myoblasts

Reactivation of G_0_ arrested undifferentiated myoblasts expectedly led to down regulation of *PAX3* and *PAX7* and up regulation of *MYF5* and *MYF6* suggesting a shift towards a proliferative cellular state where the potential to differentiate is enhanced. The expression of *MYOD1*, *MYOGENIN* and *MYH8* also dropped, probably due to loss of the small proportion of cells which responded to suspension culture by entering a differentiation pathway. These triggered cells likely did not attach to the cell culture surface and were lost. However, in the remaining majority of re-attached cells, the expression of *MYOD1* increased 32–48 hours after reactivation.

Direct tests determined that the G_0_ arrested cells were able to differentiate normally with formation of multinucleated fibers when reactivated in GM and then triggered to fuse by reduced serum. In the differentiated cultures expected increases in the expression of differentiation regulators (*MYOGENIN*, *MEF2C*, *M-CAD*) and their target structural proteins (*NCAM*, *DESMIN*, and *MYH8)* and simultaneous large down regulation of early markers *MYOD1*, *MYF5* and *MYF6* was observed. Thus, the reversibly G_0_ arrested cells were subsequently able to enter irreversible G_0_ arrest during differentiation, as expected for quiescent stem cells.

### The role of cMET, FGFR1, FGF2 and SGCA during Myoblast Activation and Differentiation

Our results indicated that cMET, the receptor for HGF, was present during G_0_ arrest and reactivation suggesting that the availability of HGF is important for participation in a myogenic response.

On contrary, both the receptor FGFR and its ligand FGF2 were highly induced during the early activation phase indicating an important role for FGF2 stimulation. Moreover, a previous study has shown that NCAM stabilizes and sustains the expression of FGFR1 [76], sustaining FGFR1 signalling. Both receptors, C-MET and FGFR1, may work at different levels of activation or work in concert to transduce cues for early activation of satellite cells.

A previous study confirmed complex formation of FGFR1 and SGCA in myogenic progenitor cells [77]. With up regulation of FGFR1 and SGCA in differentiated myofiber, such interaction may occur, however the role for SGCA during early activation of myoblasts is unclear, since only a very small portion of cells were SGCA positive while nearly all of the cells were FGFR1 positive.

### Inter Individual Differences in Primary Cultures

The use of isolated primary cells introduces inter-individual variation. Presence of such variation in the in vitro model would allow more in vivo relevant studies particularly in intervention studies; however, extensive differences between cultures might compromise firm conclusions. The origin and degree of variation is therefore of interest. Cultures A, B, and C were derived from 3 different persons and 2 different muscles**,** vastus lateralis (A and B) and gluteus maximus (C). The cultures present some similarities as well as differences in gene expression during quiescence, activation and differentiation. Mostly, gene expression patterns were similar, but the levels of expression were different for some genes. In particular, culture C showed lower expression levels of some muscle characteristic genes. However, comparing the differentiated cultures no major differences in levels between culture B and C were found concerning e.g. *MYOGENIN*, *MEF2C*, and *MYH8*, despite B otherwise showing the highest expression of the other MRFs. Comparing the two vastus lateralis cultures, *PAX3* expression was higher in A, while *PAX7* was higher in B. Further, one of the vastus lateralis-derived cultures displayed a pattern closer to the gluteus maximus-derived culture than the other vastus culture with respect to *PAX3* and *MYOD*.

The satellite cell population in different muscles are known to display differences in expression and regenerative capacity [78], bus these were not directly tested in the present study. Inter individual differences that may be responsible include direct differences in expression, and variations in growth capacity that might introduce secondary variations in gene expression. Consistent with this notion, KI67 protein expression showed that sample B had lower growth rate compared to the others. Thus, harvesting primary isolated cells based on time of culture could result in different degrees of confluence, which could introduce a difference in the myogenic status reached by the cultures. The presence of inter-individual differences would indicate some degree of preservation of the in vivo characteristics of the cells.

### Asynchronously Proliferating Cultures Compared to Synchronously Reactivated Myoblasts

Comparing microarray analysis of asynchronous (BG_0_) with synchronous cultures (AG_0_) showed approx. 4500 differentially expressed genes, underlining the distinction between long term proliferating cells compared to the reactivated short term proliferating cells. The exponentially growing long term cultures (BG_0_) though dominated by proliferating cells, represents cells in all phases of the cell cycle, and would also contain a small proportion of quiescent and differentiating cells. In contrast, reactivated cultures are synchronized in a specific cell cycle phase (depending on the time after reactivation). The reactivated cell culture (AG_0_), therefore provides a more homogeneous source for studies of proliferating myoblasts.

Our gene array results showed that pathways involved in cytokine-receptor interaction, regulation of actin cytoskeleton, focal adhesion, cell adhesion, Wnt signaling pathway, calcium signaling pathway, and ECM-receptor interactions were all significantly changed when cells passed from G_0_ to proliferation and differentiation, which reinforces the notion that the reversible G_0_ phase is sustained by interplay between many different mechanisms.

### Conclusion

We describe an in vitro model for reversible arrest of primary isolated human myoblasts in the G_0_ phase. Reactivation of G_0_ arrested cells is initiated within hours and the process occurs synchronously. The pattern of gene expression from activation to the end of the first cell cycle matched the in vivo regeneration pattern. Together with the differences in global gene expression between asynchronously proliferating cultures and reactivated synchronous cultures, these observations suggest that synchronized cultures are better models of satellite cells *in vivo*. Most myogenesis-related genes tested were expressed as reported previously during G_0_ arrest, reactivation and differentiation. We conclude that synchronized human myoblast cultures will not only permit analysis of the mechanisms involved in inducing and maintaining quiescence but also the elucidation of early activation steps. Understanding these mechanisms in cultures will assist the long-term goal of activating satellite cells in vivo for therapeutic use.
